# Mixing in Moderation: Slow Transmission of Non‐Local Macroparasites Following a Population Augmentation of an Endangered Australian Skink

**DOI:** 10.1111/mec.70121

**Published:** 2025-09-30

**Authors:** Bonnie T. Derne, Stephanie S. Godfrey, Mark N. Hutchinson, Philip Weinstein, Michael G. Gardner

**Affiliations:** ^1^ College of Science and Engineering Flinders University Adelaide South Australia Australia; ^2^ Department of Zoology University of Otago Dunedin New Zealand; ^3^ South Australia Museum Adelaide South Australia Australia; ^4^ School of Public Health/School of Biological Sciences University of Adelaide Adelaide South Australia Australia

**Keywords:** genotyping, host–parasite relationship, skink, *Tiliqua adelaidensis*, translocation, transmission, wildlife

## Abstract

Translocating threatened wildlife to more suitable habitat is increasingly necessary for conserving biodiversity. However, parasite dynamics in such translocations are poorly characterised, despite their potential importance for influencing translocation success and contributing to biodiversity and ecosystem function. We used single nucleotide polymorphism (SNP) genotyping to evaluate the transmission of parasites with different population origins following a population augmentation of the endangered pygmy bluetongue lizard (*Tiliqua adelaidensis*) involving three isolated, wild populations in South Australia. We examined inter‐population genetic variation for source and recipient host populations in the ecotoparasitic mite *Ophiomegistus michaeli* and the nematode pinworm *Pharyngodon wandillahensis*. Ordination and STRUCTURE analyses of SNP markers revealed population‐based genetic structure, particularly for *P. wandillahensis*. For 2 years following the population augmentation, hosts mostly retained parasite genotypes congruent with their origin, though cluster exceptions suggested some inter‐population transmission over time. Modelling of parasite pairwise relatedness over time supported different *P. wandillahensis* lineages gradually infecting hosts from different sources, as relatedness increased between nematodes collected from different hosts, particularly those from different source populations, and conversely decreased between nematodes collected from the same host. In contrast, *O. michaeli* pairwise relatedness changed little over time, suggesting minimal inter‐host movement. The apparently minimal and slow nature of transmission of non‐local mites and nematodes between translocated and resident host lizards is likely driven by the non‐social nature of *T. adelaidensis* and as yet uncharacterised aspects of the parasites' life histories, highlighting the importance of considering these during conservation management.

## 1 Introduction

Wildlife translocations are an increasingly used conservation tool as the world's biodiversity is threatened by habitat degradation and climate change (Armstrong and Seddon 2008; Fischer and Lindenmayer 2000; Fordham et al. 2012; Schlaepfer and Lawler 2023). However, this conservation strategy has historically been prone to failure; this is due to a range of factors (Berger‐Tal et al. 2020; Morris et al. 2021; Sheean et al. 2012), including the impact of infectious disease (Beckmann et al. 2022; Kock et al. 2010). Despite the potential negative effects of disease, little is known about the pathogens and other parasites infecting wildlife hosts, especially during translocations (Northover et al. 2019; Thompson et al. 2010). Transmission of parasites and microbial pathogens can occur in either direction between translocated animals and their recipient ecosystem (Tompkins et al. 2011). These infections can reduce the survival of already threatened translocated and/or recipient hosts; therefore, jeopardising conservation gains (Daszak et al. 2000; Dunlop and Watson 2022; Kock et al. 2010). Translocations can also disrupt existing host–parasite relationships, potentially resulting in more severe pathology and increased parasite transmission (Aiello et al. 2014; Lebarbenchon et al. 2006; Northover et al. 2018; Portas et al. 2016). Significant adverse impacts of parasites on translocations of herpetofauna are exemplified by several native frog translocations in eastern Australia, where infection by the parasitic fungus *Batrachochytrium dendrobatidis* has caused lowered survivorship or breeding at a new site, in some cases to the point of outright translocation failure (Scheele et al. 2021).

While parasites present a potential risk for translocated animals and their recipient communities, the paradigm that they are purely harmful for wildlife is proving to be an oversimplification. Growing evidence suggests that parasites are important, if understudied, components of functional ecosystems (Dunn et al. 2009; Fischhoff et al. 2020; Hasik et al. 2023; Kuris et al. 2008; Lafferty et al. 2006). Translocating parasites with their hosts may therefore help preserve ecosystem function and evolutionary potential, and thus increase translocation success in the long term. Host‐specific parasites of endangered hosts are also likely to be threatened and themselves have intrinsic conservation value (Derne et al. 2019; Dunn et al. 2009; Lymbery and Smit 2023; Northover et al. 2018; Strona 2015), and should be translocated with their hosts if adverse effects are considered unlikely following rigorous assessment (IUCN/SSC 2013; Lymbery and Smit 2023). Avoiding further biodiversity loss during host–parasite translocations requires a deeper understanding of parasite diversity and the host–parasite relationship under translocation conditions (Dunlop and Watson 2022; Hartley and Sainsbury 2017; IUCN/SSC 2013; Northover et al. 2018, 2019, 2024)—as well as how parasites are affected by host decline (Strona 2015).

Determining how host–parasite relationships are affected by translocation requires the identification of parasites, and ideally, the tracing of their transmission between the introduced individuals and the recipient community following translocation. The ability to distinguish between conspecific parasites from different populations is needed to infer transmission in the case of population augmentations (also known as reinforcements), or reintroductions involving multiple founder populations, where population augmentations and reintroductions both involve the intentional movement of animals and are therefore each a type of translocation (Seddon 2010). Genetic markers such as single nucleotide polymorphisms (SNPs) provide a powerful tool for these purposes (Archie et al. 2009), enabling confident population assignment of individual animals (e.g., Jenkins et al. 2019). For species‐specific parasites with limited dispersal ability, population‐level genetic differentiation can be more pronounced for the parasite than for the host (Cole and Viney 2018; Falk and Perkins 2013; Fricke et al. 2010; Mazé‐Guilmo et al. 2016). As such, parasite genotyping can also be a useful method of identifying a host's provenance and better understanding the demographic history of both parasite and host populations, which can inform conservation strategies for both species (Carlson et al. 2020; Criscione et al. 2006; Sromek et al. 2024; Whiteman and Parker 2005).

A number of wildlife studies have genotyped bacteria to trace intra‐population transmission (Balasubramaniam et al. 2019; Blyton et al. 2014; Bull et al. 2012; Proboste et al. 2019; VanderWaal et al. 2014; Wright et al. 2024), and others have genotyped macroparasites for transmission tracing within animal populations using microsatellites, or a region of the mitochondrial COI gene (e.g., Dharmarajan et al. 2010; Fricke et al. 2010; Lahmar et al. 2004; Neal et al. 2016). The use of genome‐wide SNP markers for parasite genotyping still appears limited in wildlife parasitology, being more common for parasites and vectors of human health significance (e.g., Amambua‐Ngwa et al. 2019; Campos et al. 2017; Diawara et al. 2013; Shrestha et al. 2024). Similarly, whilst there are studies looking at parasite communities or bacterial prevalence in wildlife translocations (e.g., Baling et al. 2013; Cheng et al. 2015; Fairfield et al. 2016; Moir et al. 2012; Northover et al. 2019; Portas et al. 2020), there have been few published studies using parasite genotyping at a sub‐species level in this context (but see Grange et al. 2016, 2017). Here, we explore the potential of SNP genotyping to better understand how parasites are transmitted among hosts following a wildlife translocation.

In this study, we conducted a wild‐wild experimental population augmentation of the endangered skink, the pygmy bluetongue (*Tiliqua adelaidensis*), within experimental enclosures. This species is endemic to South Australia and is of generally low vagility (Bull et al. 2015). We examined genetic differences among parasite populations at the two source and one recipient location, and also during the post‐translocation period to infer inter‐population transmission. To achieve this, we studied the two macroparasite species for which *T. adelaidensis* is the only known host: an oxyuroid gut nematode, *Pharyngodon wandillahensis*, and the paramegistid mite, *Ophiomegistus michaeli* (Derne et al. 2019; Fenner et al. 2008). This experimental population augmentation was the first translocation of *T. adelaidensis* in a wild setting, and provided an opportunity to elucidate parasite dynamics between hosts of different population origins when they are united by translocation. Neither *P. wandillahensis* nor *O. michaeli* have obvious fitness costs to their host (Derne et al. 2019; Fenner et al. 2008; Smith, Fenner, et al. 2009; Smith, Gardner, et al. 2009), though these costs could be subtle and as yet unmeasured (Fenner and Bull 2008; Fol and Mostafa 2021), or may be heightened by translocation‐induced stress (Benítez‐Malvido et al. 2019; Dickens et al. 2010). Furthermore, understanding transmission of known parasites may also inform effective management of future disease outbreaks by pathogens with similar transmission mechanisms.

### 1.1 Aims

In order to better understand parasite transmission dynamics following a population augmentation of *T. adelaidensis*, our aims were to:

- Determine how genetically differentiated *P. wandillahensis* and *O. michaeli* are among geographically isolated host populations. We expected that these parasites would show strong population‐level genetic structure since their host has low vagility and demonstrates significant inter‐population genetic differentiation (Smith, Gardner, et al. 2009).
- To determine if nematodes and mites of non‐local origin were transmitted among lizard hosts from three different populations of origin once combined in a population augmentation. We predicted that changes in parasite genotype, reflecting the transmission of non‐local parasites, would occur among lizards in the same enclosure throughout the initial two‐year post‐translocation period. However, we hypothesised that this genetic change representing transmission would be gradual, due to the low vagility and solitary nature of *T. adelaidensis*.

## 2 Materials & Methods

### 2.1 Study System

*Tiliqua adelaidensis* is a medium scincid lizard with an unusual ecology; it obligately occupies spider burrows (dug by certain mygalomorph and lycosid species) in mesic grassland patches within a small region of southern Australia (Fenner et al. 2018; Hutchinson et al. 1994). Among isolated populations, there is low to moderate, but significant, genetic differentiation, particularly between southern and northern population groups more than 70 km apart (Smith, Gardner, et al. 2009). Whilst anthropogenic activities have severely fragmented the grassland habitat of this species (IUCN 1996; Lunt 1998), the inter‐population genetic differentiation observed may predate modern settlement (Smith, Gardner, et al. 2009). Low vagility is thought to drive the genetic structure observed over distances greater than 30 m in even continuous habitat (Schofield et al. 2014; Smith, Gardner, et al. 2009). This endangered skink is further threatened by climate change, making translocations to more suitable habitat a necessary conservation strategy in the coming decades (Fordham et al. 2012).

As an oxyurid (pinworm) nematode, *P. wandillahensis* has a single‐host, direct lifecycle, and exhibits haplodiploidy (Adamson 1989). Transmission of *P. wandillahensis* to new *T. adelaidensis* hosts presumably occurs by ingestion of faeces containing infective, non‐motile eggs, with development and reproduction taking place in the host's gut (Adamson 1989), though the length of the lifecycle in this species (and congeners) is unknown. Prevalence within a host population has been found to be 0%–34% (Fenner et al. 2008; Smith, Fenner, et al. 2009). Fenner et al. (2011) reported that *T. adelaidensis* individuals which had burrows close to ‘disperser’ individuals (i.e., ones that stayed in the study area for less than 2 months) had higher nematode prevalence than other lizards. The authors hypothesised that the observed tongue‐flicking at scats of unfamiliar individuals for social information (Fenner and Bull 2010, 2011) may provide a transmission pathway. Prevalence of *O. michaeli* in *T. adelaidensis* varies considerably (between 0% and 46%) among populations, and even within habitat patches less than 100 m wide, as well as with time of year (Derne et al. 2019). The non‐adult life stages and transmission mechanisms of *O. michaeli* and congeners are unknown, though *T. adelaidensis* burrows may provide suitable conditions for any free‐living life stages to persist and for new hosts to become infested if they share a burrow asynchronously (Derne et al. 2019).

### 2.2 Experimental Population Augmentation (Translocation)

**FIGURE 1:**
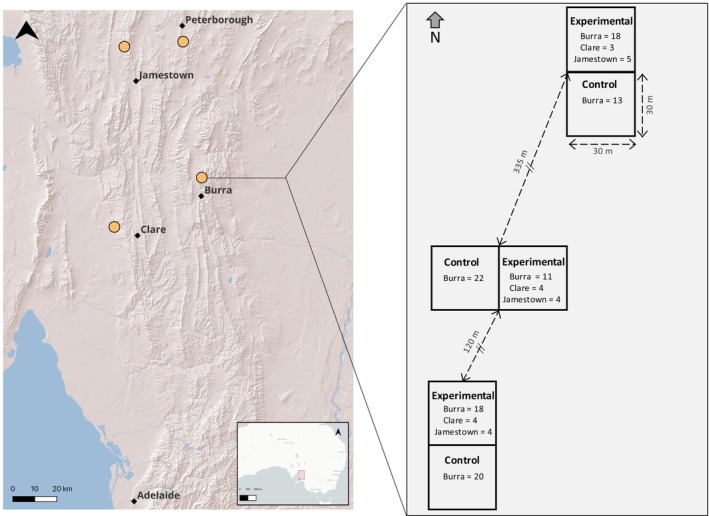
Approximate locations of the four *Tiliqua adelaidensis* populations (orange points) sampled for parasites in this study, with nearby townships used to name these populations. The right‐hand panel represents the 30 × 30 m enclosures containing indicated numbers of *T. adelaidensis* individuals that were part of the experimental population augmentation at the release site near Burra, South Australia.

The experimental set‐up and population augmentation that this study is based on are outlined by Clive et al. (2020). All work conducted was approved by the Flinders University Animal Welfare Committee (permit E417/15 & E45/17) and the South Australian Department of Water and Natural Resources (permit G25011‐12). Briefly, three pairs of 30 × 30 m fenced enclosures (pairs were approximately 120–335 m apart) were erected around established *T. adelaidensis* individuals in a wild population on a livestock‐grazed grassland north‐east of Burra township, South Australia in July 2015 (Figure 1). One enclosure in each of the three pairs was designated as a control enclosure, whilst the other, adjoining, enclosure in the pair was designated as an experimental enclosure. In February 2016, 11 adult or subadult individuals were captured from another wild *T. adelaidensis* population west of Clare township, and 13 individuals were captured from a wild population north of Jamestown. These individuals were translocated to the Burra site and were released into artificial dowel burrows in the experimental enclosures, amongst the established Burra resident conspecifics. Each of these three experimental enclosures contained a mixture of individuals from different population origins: 11–18 resident Burra individuals, 3–4 translocated Clare individuals, and 4–5 Jamestown individuals (Figure 1). The three control enclosures each contained 13–22 Burra residents only; no translocated individuals were added to these (Figure 1).

### 2.3 Sample Collection

Over one fortnight per month from October 2015 to March 2016, October 2016 to March 2017 and from October 2017 to March 2018, the capture of all individuals in the six enclosures was attempted by identifying lizard‐occupied burrows and luring lizards out with tethered bait (Milne 1999). Typically, less than 10% of lizards in an enclosure remained uncaught for each month. In January–February of 2016, 2017 and 2018, neonates were captured within 3 weeks after birth (in addition to the 126 individuals cited above) and reared captively as part of a concurrent study (Clive et al. 2020). Individual lizards were identifiable by the specific toe clip sequence given at first capture toes were retained for DNA analysis for a parallel study (Clive et al. 2020). This method of marking has been routinely used for this species and other skinks due to the unsuitability of other marking methods, with no known adverse effects on behaviour or survival (see Langkilde and Shine (2006)). Upon initial and each monthly capture thereafter, faecal pellets were collected if a lizard defecated in the approximately 10 min it was held. From February 2016 onwards, each lizard captured at Burra was also examined for adult mites. If mites were evident, at least one mite was collected from an individual per capture occasion, whilst one or more were left in situ so as to preserve the host–parasite relationship. Both scats and mites were immediately placed in 95% ethanol, which was stored at 4°C from the end of each day.

Mites and nematodes were also collected from non‐translocated hosts in the Jamestown population between October 2016 and March 2018, in addition to mites and nematodes from Burra resident lizards and the translocated Clare and Jamestown lizards involved in the experimental population augmentation. Further sampling at the Clare population was not possible due to a lack of on‐going access to the private property. To assess broader population separation, nematodes were also collected from another *T. adelaidensis* population which was not involved in the experimental translocation, located south of the Peterborough township between January and March 2018 (Figure 1).

After 1–6 months of storage in ethanol at 4°C, each lizard scat was examined under a dissecting microscope, and adult and subadult nematodes were separated from the other material and stored in 95% ethanol. All nematodes had the same macroscopic appearance (colour, shape, size range) and were assumed to be *Pharyngodon wandillahensis*, the only nematode species known to parasitise *Tiliqua adelaidensis*. Each individual mite and nematode had the host individual, location, and date of collection recorded. Subsets of 120 individual mites and 189 nematodes were selected for genotyping. These genotyped mites and nematodes represented all occasions where an infested host was captured in an enclosure at Burra, and most mites and nematodes collected from the Jamestown and Peterborough sites.

### 2.4 Single Nucleotide Polymorphism Identification and Analysis

The laboratory and data analyses are described in further detail in the Supporting Information section, and are briefly outlined here. Extraction, restriction enzyme digestion (using PstI and SphI), and Next Generation DNA sequencing and SNP calling from whole nematodes and mites were conducted using proprietary protocols and software developed by Diversity Array Technology Pty Ltd. (DArTseq) (Kilian et al. 2012). This methodology is similar to ddRADseq, though with its own, reproducible approach to library preparation and fragment selection, and has been successfully used to answer a range of population genetic questions in non‐model animal species (Gruber et al. 2018; Melville et al. 2017). Single nucleotide polymorphisms (SNPs) identified underwent a series of filtering steps, which were carried out with the R package ‘dartR’ (Gruber et al. 2018). Filtering consisted of first removing individuals and loci for which the reproducibility (averaged over two allelic states) fell below 100%, and removing monomorphic loci. Loci that had over 25% missing data across individuals or within individuals were removed, and then any secondary loci on sequence tags were removed. Loci pairs with a Hamming distance of less than 0.2 were removed to reduce the possibility of sequencing error being confused with a different locus. Filters to remove any loci not in Hardy–Weinberg equilibrium were applied. Loci in linkage disequilibrium were retained in the interests of retaining the maximum number of SNPs for population‐level differentiation. An additional filter to remove loci with minor allele frequencies less than 1% was applied for subsequent relatedness analysis. Although contamination from other DNA (e.g., arising from consumption of host gut content, faecal content, other unknown nematode species, or blood meal) cannot be definitively ruled out, stringent filtering of loci with less than 100% sequencing reproducibility and loci and individuals with more than 25% missing data is expected to have minimised the potential effect of any contamination. The called SNPs were not aligned to any whole genome for annotation purposes due to the lack of available reference genome from any species within either Pharyngodonidae (for *P. wandillahensis*) or Paramegistidae (for *O. michaeli* ).

In order to examine genetic differentiation of mites and nematodes among host populations, three analytical approaches were used to group individuals according to genetic similarity: principal coordinates analysis (PCoA), discriminant analysis of principal components (DAPC), and Bayesian cluster analysis carried out with STRUCTURE. Genetic similarity between individual parasites from different populations was first examined using PCoA (Gower 1966) in dartR, where individuals were entities, and the genotypes of SNP loci were attributes. A second non‐model‐based method for grouping individuals based on genetic similarity (which maximises inter‐group variation rather than the inter‐individual variation of PCoA) was applied by performing cluster identification and DAPC with the R package ‘adegenet’ (Jombart et al. 2008). This was done by transforming data into principal components (PCs) and retaining these for initial cluster identification. A biologically plausible number of clusters that maximised variation (K) was selected using the Bayesian Information Criterion (BIC). DAPC was then performed with the most informative PCs only, to avoid model overfitting and instability of membership probabilities (Jombart and Collins 2015). All discriminant functions were retained since the number of possible clusters was low.

The software STRUCTURE 2.3.4 (Pritchard et al. 2000) was then used to conduct Bayesian model‐based cluster analysis, as an alternative method of examining whether or not SNP genotypes clustered by host population of origin. Using GNU Parallel (Tange 2018), 20 replicates for each value of k (number of populations) between 1 and 10 were run with different random seeds, as recommended by Evanno et al. (2005). Each run consisted of 100,000 burn‐in iterations as deemed sufficient by the convergence of values summary (Porras‐Hurtado et al. 2013) and 100,000 Markov Chain Monte Carlo (MCMC) repetitions. The population‐specific prior was selected (POPALPHA = 1) and alpha was set to 1/K, where K was the assumed number of populations (Wang 2017). All other extra parameters were left at default options; notably, admixture models were used, and the correlated allele frequencies option was selected (Falush et al. 2003). In the absence of a genetic map, linkage models were not used.

The most likely number of groups was identified using the Evanno method (Evanno et al. 2005) in STRUCTURE HARVESTER (Earl and VonHoldt 2012) after examination of the log‐likelihood plot to ensure the real number of groups was more than one. The estimated membership probabilities of different clusters for each individual were identified with CLUMPAK using the greedy algorithm (Kopelman et al. 2015) and visualised with DISTRUCT (Rosenberg 2004). When two or more likely clusters were identified by STRUCTURE, each cluster was further hierarchically analysed using the steps described above in order to identify all clusters rather than just those at the highest level of hierarchy (Coulon et al. 2008; Evanno et al. 2005; Janes et al. 2017). Sub‐clusters of less than four individuals identified during hierarchical analysis were considered as one cluster and not further analysed (Shi et al. 2010).

### 2.5 Relatedness

Pairwise relatedness among mite individuals and among nematode individuals was calculated from SNP genotypes with the aim of examining parasite transmission among hosts within enclosures. Mite pairwise relatedness was calculated using the dyad maximum likelihood estimator (Milligan 2003), implemented by the program COANCESTRY 1.0.1.9 (Wang 2011). This estimator was selected because it showed the highest correlation with the simulated dataset (Wang 2011) (see Table S1). Pairwise relatedness for the nematode *P. wandillahensis* was calculated using the Ritland's estimator of relatedness with Huang's correction (Huang et al. 2015). This calculation was implemented in the program PolyRelatedness 1.8 (Huang et al. 2014) to account for haplodiploidy exhibited by oxyuroid nematodes (Adamson 1989). Relatedness in both the mite and nematode calculated using comparisons between all individuals was then subsetted to only include relatedness between pairs of individuals that were found in the same enclosure.

In order to understand how genetic relatedness patterns in nematodes and in mites changed over the 2 years following the translocation of their hosts (as an indicator of transmission of parasites from different host sources), we constructed three separate linear mixed models for each of nematode and the mite datasets. To track the extent of parasite transmission and resulting genetic change, these models examined the effects of host type (same host individual or different host individual) and host origin over time following translocation (as fixed effects) on parasite pairwise relatedness as the response variable. Parasite individual identity, host identity, and enclosures were included as random effects in order to account for sources of non‐independence. Nematode pairwise relatedness was normally distributed; therefore, linear mixed models were constructed with the lme4 and car packages (Bates et al. 2015; Fox and Weisberg 2019) in the R environment (R Core Team 2022). Mite pairwise relatedness data were highly skewed, so generalised linear mixed models (Gamma distribution with a log link) were constructed using the glmmTMB package (Brooks et al. 2017) in the R environment. To account for the zero‐inflated nature of mite relatedness values, 0.001 was added to all values.

Firstly, to determine the effect of time elapsed between collection of each member of a parasite pair on their relatedness, we fitted a model for each parasite species where the fixed effects were the number of days between collection within the same season, as well as whether members of a pair were collected from the same host individual versus a different host individual (See Tables S2 and S3). To account for any effect of time between collection on pairwise relatedness, we then limited our focal analyses to relatedness between mites or nematode pairs collected within the same 30 day period. We omitted one outlying relatedness value for the only two successfully genotyped mites collected in the same 30 day window during the final (2017–2018) field season from these analyses. To examine how relatedness of parasites from the same host changed over the post‐translocation study period, the first of these models included fixed effects for whether the nematodes or mites in a pair came from the same or different host individuals, and time since translocation. Finally, for parasite pairs collected from different hosts, we focused on how host origin affected relatedness following translocation. These second models included host origin (same or different) and time since translocation as fixed effects. The ggpredict function from the ggeffects package (Lüdecke 2018) was used to generate predicted relatedness values for each model.

## 3 Results

During the population augmentation study (October 2015–March 2018), 126 *T. adelaidensis* individuals (102 from Burra, 11 translocated from Clare, and 13 translocated from Jamestown) were captured within the enclosures at least once over 18 spring–summer monthly sampling trips. There were 996 capture events during this period, where the median number of capture events per individual was eight (interquartile range: 9). Of these, 828 capture events occurred after the population augmentation (March 2016–March 2018). Nematodes were visually detected in the scats of 59 Burra‐originating individuals (52.5% of adult/sub‐adults, 10.16% of juveniles < 1 year old), four individuals translocated from Clare (2.75%), and four (3.25%) translocated from Jamestown. Mites were detected on seven Burra individuals (6.9% of adult/sub‐adults, 1.6% of juveniles < 1 year old), two individuals translocated from Clare (5.5%), and eight lizards translocated from Jamestown (61.5%). The majority of offspring born in January and February of 2016, 2017, and 2018 were sampled and removed from the enclosures shortly after birth. However, some offspring evaded detection until the next field season and were either left in situ and sampled along with older sub‐adults and adults if from an experimental enclosure (*n* = 6) or placed in suitable habitat outside the enclosures if they were from control enclosures and never recaptured (*n* = 16). One mite and seven nematodes genotyped in this study were from six such offspring, all from control enclosures. Additional sampling of lizards not involved in the translocation detected nematodes from four Jamestown individuals (11.4% of individuals captured at least once) and two Peterborough individuals (8.3%), and mites from 15 Jamestown individuals (42.8%).

### 3.1 Genetic Population Differentiation

DArTseq sequencing produced genotypes for 118 of 120 *Ophiomegistus michaeli* mite individuals submitted, consisting of 6736 binary SNPs with 42.7% missing data. Filtering steps resulted in 102 genotypes, with 458 SNPs and 10.68% missing data. Filtering for minor allele frequencies of less than 1% for relatedness analysis further reduced the number of SNPs to 442 loci. For *Pharyngodon wandillahensis* nematodes, DArTseq sequencing produced 184 genotypes (of 189 individuals submitted for sequencing) and 2478 binary SNPs with 50.78% missing data. Filtering steps produced 147 genotypes with 395 SNPs and 3.18% missing data. Filtering for minor allele frequencies of less than 1% for relatedness analysis reduced the number of SNPs slightly, yielding genotypes with 358 loci.

**FIGURE 2:**
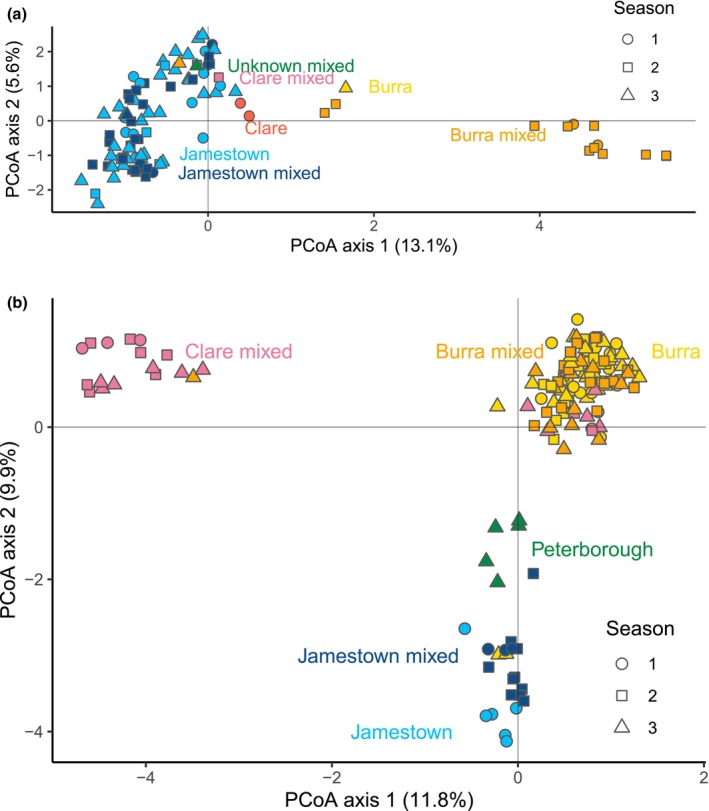
Principal Coordinates Analysis representing genetic variation in macroparasites collected from *Tiliqua adelaidensis* hosts over three spring–summer seasons. Collection periods (seasons) are denoted by point shape, with colours corresponding to host population group. ‘Burra’ individuals (shown in yellow) are from Burra resident lizards in control enclosures not exposed to translocated conspecifics. ‘Jamestown’ individuals (shown in pale blue) were collected from Jamestown lizards that were either not translocated to Burra, or translocated to Burra after mite collection. ‘Clare’ parasites (shown in red) were sampled from a Clare host immediately prior to translocation to Burra. ‘Clare mixed’ (pink), ‘Jamestown mixed’ (dark blue) and ‘Burra mixed’ (orange) refers to parasites from experimental enclosures following the translocation, where lizard hosts originating from three populations were present. Axes lengths are proportional to the variation they represent. (a) Variation among 102 *Ophiomegistus michaeli* (mite) genotypes. ‘Unknown mixed’ (shown in green) is one individual found in an experimental enclosure where the host was not recorded. (b) Variation among 147 *Pharyngodon wandillahensis* (nematode) genotypes. ‘Peterborough’ nematodes (shown in green) were collected from the Peterborough population which was not involved in the translocation.

The genotype‐based grouping patterns that were inferred from PCoA, DAPC, and STRUCTURE analysis of *O. michaeli* individuals were broadly consistent with each other and revealed genetic differentiation among isolated host populations (Figure 2a, Figures S4 and S5). The results showed general clustering of mites in ordination space according to their population, with mites from Jamestown lizards clustering strongly. Mites from Clare and Burra lizards demonstrated more genetic variability, with some mites from Clare and Burra clustering with or adjacent to Jamestown‐originating mites, and another distinct cluster of mites from Burra lizard hosts clustering distinctly from others in ordination space (Figure 2a, Figures S4a and S5a). A notable incongruence between genetic cluster membership and host population origin was a mite collected from a Burra‐originating lizard 20 months following translocation which clustered with mites from Jamestown (Figure 2a).

By contrast to the mites, the PCoA of *P. wandillahensis* nematode genotypes showed three distinct genetic clusters, each corresponding to the geographic origin of their host individual (Figure 2b). As with the mites, clusters revealed by PCoA were consistent with those identified by the DAPC (Figure S9) and also STRUCTURE analysis (Figure S10). While nematode genotypes did not generally appear to change in the two sampling seasons post‐translocation, some exceptions were observed. These incongruences included seven nematodes collected from Clare‐originating hosts in experimental enclosures 9–20 months after translocation, which grouped with Burra‐originating nematodes. Conversely, one nematode from the Clare genetic cluster was found in a Burra host from an experimental enclosure 20 months after translocation. Two nematodes that fell within the Jamestown genetic cluster were collected from a Burra host from a control (non‐mixed) enclosure 22 months after the translocation (Figure 2b).

### 3.2 Genetic Relatedness Patterns

**FIGURE 3:**
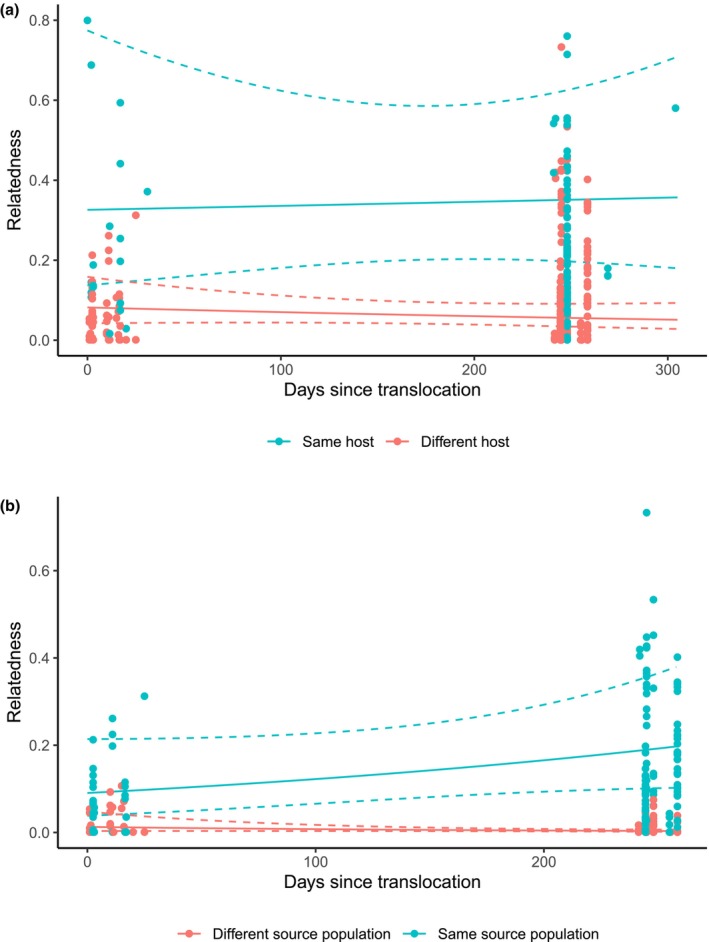
Mean predicted relatedness between *Ophiomegistus michaeli* mite pairs collected within a 30 day period, as a function of host type following a population augmentation (translocation) of *Tiliqua adelaidensis* hosts. Raw relatedness values are also included as points. (a) Predicted mite relatedness between mites collected on the same host lizard vs. a different host lizard. (b) Predicted mite relatedness between mites collected from different host lizards either from the same population of origin or from a different population of origin.

When considering all mites collected within 30 days of each other, only host identity was a significant predictor of relatedness, with significantly higher relatedness between mite pairs that were collected from the same host individual than between mites collected from different hosts (*χ* ^2^ = 47.95, df = 1, *p* < 0.001) (Figure 3a; Table 1). Time since translocation had no effect on the relatedness of mites (*χ* ^2^ = 0.40, df = 1, *p* = 0.527), regardless of whether they were from the same host or different hosts (*χ* ^2^ = 0.637, df = 1, *p* = 0.42).

For mite pairs collected from different host individuals only, those collected from lizards from the same source population were significantly more related to each other than pairs from different host source populations (*χ* ^2^ = 343.75, df = 1, *p* < 0.001). The time since translocation alone had no significant effect on the relatedness patterns of mites (*χ* ^2^ = 0.57, df = 1, *p* = 0.45), though mite pairs from the same host source appeared to be more related to each other than average at 8–9 months after translocation, while mites from a different host source did not (*χ* ^2^ = 7.94, df = 1, *p* < 0.01) (Figure 3b; Table 1).

**TABLE 1:** Analysis of deviance with Type II Wald chi‐square tests of two generalised linear mixed models (using gamma distribution with a log link) examining *Ophiomegistus michaeli* pairwise relatedness as a response to host identity (same host or different host), host origin, and time since translocation. For both models, pairwise mite relatedness is limited to mites collected within 30 days of each other. Random factors for each model are: Parasite individual identity, host individual identity.

Model focus	Fixed effects	*χ* ^2^	Df	Pr (> *χ* ^2^)
Effect of host type and time since translocation	Same/different host	47.946	1	< 0.001*
	Days since translocation	0.401	1	0.527
	Same/different host: Days since translocation	0.637	1	0.425
Effect of host origin and time since translocation	Same/different host origin	343.753	1	< 0.001*
	Days since translocation	0.570	1	0.450
	Same/different host: Days since translocation	7.937	1	< 0.01*

* Denotes a significant *p* value (alpha = 0.05).

**FIGURE 4:**
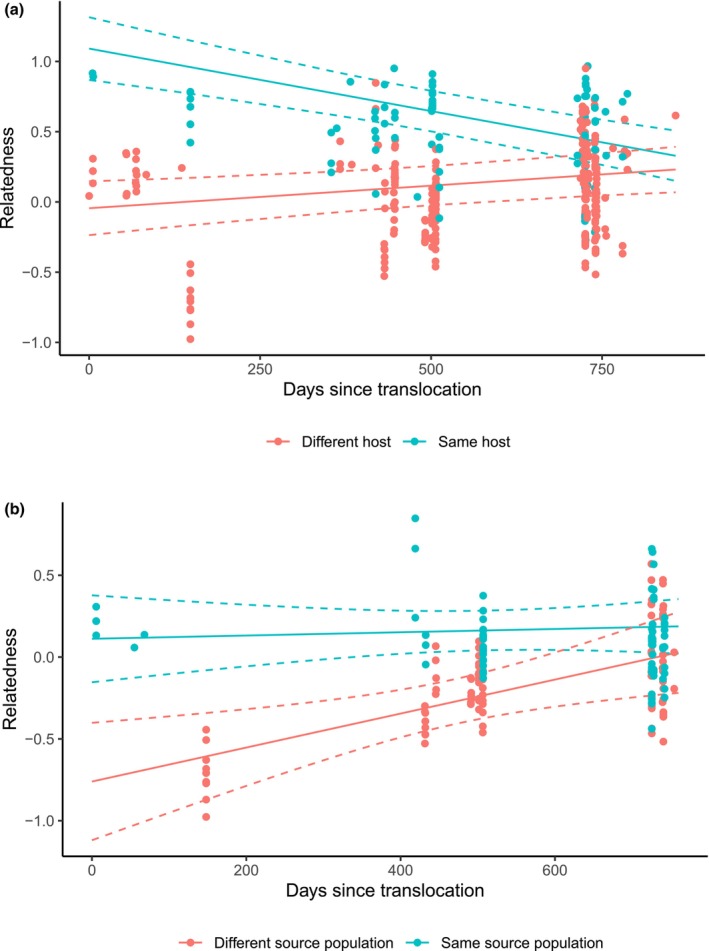
Mean predicted relatedness between *Pharyngodon wandillahensis* nematode pairs collected within a 30 day period, as a function of host type following a population augmentation (translocation) of *Tiliqua adelaidensis* hosts. Raw relatedness values are also included as points. Note that for Ritland's estimator of relatedness with Huang's correction, a value of 0 indicates a level of relatedness expected by chance. (a) Predicted relatedness between nematode pairs collected on the same host lizard vs. a different host lizard. (b) Predicted relatedness between nematodes collected from different host lizards either from the same source population or from a different source populations.

When considering nematode pairs collected within the same 30 days of each other, nematodes collected from the same host individuals were more highly related to each other than nematodes collected from different host individuals (*χ* ^2^ = 52.89, df = 1, *p* < 0.001, Figure 4a; Table 2). This difference in average relatedness was largest immediately following the translocation, and over time decreased as same‐host nematode pairs became less related, and different host nematode pairs became more related. Similarly, when only considering nematodes collected from different hosts, nematode relatedness was higher when the pair of nematodes were collected from hosts of the same origin than when collected from host individuals of different source populations at the time of translocation (*χ* ^2^ = 5.25, df = 1, *p* = 0.022, Figure 4b; Table 2). However, relatedness increased between nematodes of different origins over the two‐year post‐translocation period, eventually converging with the mean relatedness between nematodes from hosts of the same source population (Figure 4b; Table 2).

**TABLE 2:** Analysis of deviance with Type II Wald chi‐square tests of two linear mixed models examining *Pharyngodon wandillahensis* pairwise relatedness as a response to host identity (same host or different host), host origin and time since translocation. For both models, pairwise nematode relatedness is limited to nematodes collected within 30 days of each other. Random factors for each model are: Parasite individual identity, host individual identity.

Model focus	Fixed effects	*χ* ^2^	Df	Pr (> *χ* ^2^)
Effect of host identity and time since translocation	Same/different host	173.212	1	< 0.001*
	Days since translocation	0.174	1	0.676
	Same/different host: Days since translocation	52.888	1	< 0.001*
Effect of host origin and time since translocation	Same/different host origin	31.442	1	< 0.001*
	Days since translocation	3.191	1	0.0740
	Same/different host: Days since translocation	5.246	1	0.022*

* Denotes a significant *p* value (alpha = 0.05).

## 4 Discussion

Translocations are becoming increasingly common to mitigate increased habitat fragmentation and climate change. There is a general paucity of knowledge on the interactions of pathogens and other parasites infecting wildlife hosts, and particularly how these are affected by translocations (Lymbery and Smit 2023; Northover et al. 2018, 2024). As we conducted an experimental population augmentation involving three populations of the endangered skink *T. adelaidensis*, we aimed firstly to determine whether their macroparasites at the source locations showed population‐level genetic structure. Our results show that both *Ophiomegistus michaeli* mites and *Pharyngodon wandillahensis* nematodes display genetic differentiation among the geographically separated host *T. adelaidensis* populations that we surveyed. We next asked whether source population‐specific parasite genotypes changed following the population augmentation, consistent with parasite transmission among lizard hosts from different sources. As we hypothesised, there were changes in parasite genotypes and relatedness following the population augmentation, suggesting that transmission of source population‐specific genotypes occurred among hosts from different sources. These changes occurred after several months and were very limited in the case of *O. michaeli* . Comparison of parasite relatedness within and among hosts and host groups of different origins showed at least an initial tendency for hosts to retain a parasite genotype consistent with their source population. For *P. wandillahensis*, however, these different lineages gradually spread through a mixed‐source host population and led to genetic homogenisation over the 2 years following translocation. The inter‐population genetic structuring observed for these poorly‐characterised parasites suggests some degree of host‐species specificity and expected low vagility, and relatedness patterns indicate that successive parasite generations may infect the same host individual. In using genome‐wide markers to genotype parasites collected at the time of translocation and for 2 years thereafter, these results offer unprecedented insight into how genetically distinct parasites are introduced and subsequently transmitted in non‐social host species as a result of active conservation management. This slow and limited transmission also provides a basis for speculating that potential pathogens are unlikely to spread through a population of this nature quickly following intentional introduction.

### 4.1 Parasite Genetic Structure Reflects Host Populations

Genetic differentiation among host populations was observed in both *O. michaeli* mites and *P. wandillahensis* nematodes (Figure 2), and cluster membership of individual parasites was consistent across all analysis methods (Figures S4, S5, S9 and S10). Genetic differentiation in these parasites was expected, given that a prior analysis of *Tiliqua adelaidensis* microsatellites found a low to moderate, but significant, amount of genetic differentiation among populations as little as 1.7 km apart (Smith, Gardner, et al. 2009). Furthermore, genetic differentiation observed between isolated populations of *P. wandillahensis* is consistent with the biology of oxyuroid nematodes, though there appears to be little known beyond identified host species for the genus *Pharyngodon* and the broader family (Fol and Mostafa 2021). Species in the order Oxyurida tend to be host‐specific, which limits gene flow over spatial scales, particularly if the host has low vagility (Adamson 1989; Falk and Perkins 2013). Adding to their low dispersal ability is the fragile nature of the infective egg stage, which is desiccation‐ and temperature‐sensitive and thus may not persist for long outside of the host (Adamson 1989). Haplo‐diploidy exhibited by this group also contributes to high levels of inbreeding (Adamson 1989, 1990). Inter‐population genetic structure appeared weaker for the *O. michaeli* mites (Figure 2). Similarly to the nematode species, little is known about the biology of the paramegistid genus that *O. michaeli* belongs to, though a few species have been recorded on more than one skink or snake host species (Klompen and Austin 2007), and this weaker interpopulation genetic structuring may hint at the use of another host species by *O. michaeli* . It is speculated that scale size may preclude the mites from exploiting smaller skinks and snakes however; presumably this would increase host specificity at a local scale (Derne et al. 2019). More extensive surveying and sampling of this apparently rare mite species across more host populations may improve our ability to characterise genetic structure.

### 4.2 Limited Transmission for Non‐Local Parasite Genotypes

When mite and nematode parasites of lizards involved in the population augmentation were examined by ordination and clustering analyses, the majority remained within the same genetic clusters as parasites from untranslocated hosts of the same origin throughout the study. This finding suggests that parasite genotypes of a given host remained largely unchanged in the 2 years following the population augmentation (Figure 2). However, the few exceptions to this trend provide strong evidence that transmission of parasites with non‐local genotypes following the population augmentation did occur, albeit to a limited extent. Modelling parasite pairwise relatedness over time provided a more sensitive measure of genetic change. At the point of translocation, nematodes from the same host individual or same host origin had higher relatedness than those from different host individuals or hosts from different origins, reflecting the population differentiation observed in the ordination and cluster analyses. However, over time, the predicted relatedness of nematodes collected from different hosts and hosts of different source populations increased, converging with relatedness of nematodes from the same host or same host origin towards the end of the 25‐month study period (Figure 4). This result suggests that transmission of nematodes between different host individuals (including those from different origins) at the translocation site occurred gradually. By contrast, predicted mite pairwise relatedness did not significantly change over the first year post population augmentation when mites on the same host vs. a different host were compared (Figure 3a), a result consistent with lizard hosts mostly retaining mites genetically similar to those collected at earlier time points. The predicted small but significant increase in mite relatedness for same‐source hosts over time (Figure 3b) also suggests minimal to no inter‐host transmission, though the low number of successfully genotyped mites (*n* = 2) found in the third field season prevented us from commenting on this relationship over a longer time frame.

The temporal dynamics of parasite transmission observed in this translocation are comparable with those observed by Northover et al. (2019) in a wild–wild population augmentation of the small marsupial *Bettongia penicillata* . Similarly to our *P. wandillahensis* result, they observed a convergence in parasite infra‐community between resident and translocated animals, though these community changes were greatest within the first few months of translocation. The formation of new population‐specific endoparasite associations (cestodes and coccidia) did take a longer period of 6 to 12 months, however. Collectively, this and our study point to the variability and extended nature of the time frame for the community–or intra‐species homogenisation of parasites following population augmentations. As translocations continue to comprise an important part of our collective conservation toolbox, further research is needed to identify any broad trends, though other wildlife translocation studies involving only founder populations have reported a range of outcomes in parasite prevalence at recipient sites after one or more years. These outcomes included complete failure to persist, decline, or maintenance of parasites (Fairfield et al. 2016; Moir et al. 2012; Portas et al. 2016), or, in the case of a Bettong translocation in eastern Australia, the formation of novel parasite associations (Portas et al. 2016).

### 4.3 Mechanisms of Parasite Transmission

Whilst we have provided a novel insight into parasite transmission dynamics following a translocation for this system, transmission mechanisms for these two parasites remain unconfirmed. The slow or extremely limited transmission patterns inferred for nematodes and mites respectively in this study, however, may reflect parasite reliance on host animals and their behaviours for dispersal, in contrast to passive dispersal into the environment. Auto‐infection as a mode of transmission may explain the retention of nematode and mite genotypes that we observed in hosts from all three origins following translocation. Some oxyuroid nematode species produce thinner walled eggs which remain in the host gut (promoting self‐reinfection), instead of passing out in faeces to potentially be ingested by a new host individual (Adamson 1989). Additionally, intraspecific interference competition reducing subsequent infection by conspecifics, as appears to occur in other nematode taxa (Adamson et al. 1992; Zervos 1988), may have contributed to the apparently slow rate of nematode transmission. It is expected that haplodiploidy in this species would also slow observed genetic change between generations, perhaps partially obscuring evidence of inter‐individual transmission.

The lack of significant change in predicted *O. michaeli* mite relatedness observed for same‐host pairs as well as the small increase in relatedness between pairs from different host individuals from different population origins (Figure 3) suggests a transmission ecology that is more strongly single‐host focused than *P. wandillahensis* nematodes. Furthermore, no sustained changes in mite prevalence were observed over the 2 years following translocation (Derne et al. 2019). We have previously hypothesised that the eggs and immature stages of the *O. michaeli* lifecycle may occur in the *T. adelaidensis* burrow and permit successive generations to attach to the same host animal (Derne et al. 2019), and the minimal change in relatedness we observed is consistent with this dynamic.

The slow change in parasite genotypes and relatedness over time observed here is also consistent with the non‐social nature of their host species, a factor that should be considered when assessing the disease risk of translocations. *Tiliqua adelaidensis* individuals are known to avoid direct interaction with conspecifics, with the exceptions of mating and also mother‐offspring contact (Milne et al. 2002; Schofield et al. 2014). Rather, the observed use of scat piles as social signals (Fenner and Bull 2011) has been hypothesised and supported by network analysis to enable *P. wandillahensis* transmission (Fenner et al. 2011). Host interactions such as mating, asynchronous burrow sharing, or burrow proximity may also create transmission opportunities for these two macroparasites, though the small sample sizes precluded further analysis. Given the evidence of post‐translocation transmission we have observed, whatever the mechanism, we predict that the admixture of *P. wandillahensis* lineages continued over the ensuing activity seasons and subsequent generations of *T. adelaidensis* hosts, noting that these skinks are capable of breeding in their second spring season (Milne 1999). Further admixture is also the likeliest outcome for *O. michaeli* mites in this population augmentation context, though we predict that this would occur over a longer time frame.

### 4.4 Implications for Future Translocations

Whether by virtue of low parasite dispersal, non‐social host‐driven transmission, or through possible local adaptation (whereby parasites are more successful at infecting local hosts relative to non‐local hosts), or a combination of these factors, our results suggest that macroparasite species can be slow to spread through a host population following a translocation. The lack of widespread and rapid transmission observed here would be likely to enable the identification of pathogen emergence (given adequate monitoring) before it affected a large proportion of the population in future translocations of *T. adelaidensis*, or hosts with a similar ecology. The slow to extremely slow nature of parasite transmission revealed in this study suggests that in this system and ecologically similar systems, translocated individuals may have time to recover from the shorter‐term stress of translocation, i.e., the post‐release effect *sensu* Armstrong et al. (2017) before being exposed to novel parasite genotypes, which may minimise fitness costs. Conversely, the slow rate of transmission emphasises the importance of extended post‐translocation monitoring (Ewen and Armstrong 2007; Germano and Bishop 2009; Northover et al. 2019), since the effects of non‐local parasites may not be evident immediately following translocation. Our findings may be particularly relevant for taxa such as the critically endangered grassland earless dragon species *Tympanocryptis lineata* , *T. pinguicolla* , *T. mccartneyi*, and the endangered *T. osbornei*. These species also inhabit spider and other invertebrate burrows in highly threatened, fragmented grasslands in south‐eastern Australia, and translocations and restoring habitat connectivity will comprise an important part of the conservation strategy for them (Commonwealth of Australia., 2023). While no parasites appear to have been documented in *Tympanocryptis* spp., it is extremely likely they host nematodes and other parasites which would be translocated with their hosts.

Like the parasite dynamics observed by Northover et al. (2019) following a woylie population augmentation, we have provided evidence that parasite transmission following a translocation can be a gradual process. However, the different dynamics observed in two species parasitising *T. adelaidensis* underline the importance of considering both parasite factors and host factors when predicting the extent and speed of parasite or pathogen transmission following a translocation (Lymbery and Smit 2023). *Tiliqua adelaidensis* provides a typical example of a species in need of active management such as translocation; a narrowly distributed host species in habitat that is specific, fragmented and sensitive to climate and anthropogenic changes. Furthermore, this skink is also an example of where previous research has given us relatively detailed understanding of the host species biology and needs in order to develop sound translocation protocols. Our findings here, however, highlight that, even for a well‐studied translocation candidate, their parasites still represent a knowledge gap that hampers disease risk assessment that has been widely called for by conservation scientists (Dunlop and Watson 2022; Ewen et al. 2015; Sainsbury and Vaughan‐Higgins 2012). How quickly a parasite reproduces, its lifecycle, and which environmental and host‐related factors are important for its spread to new host individuals are generally poorly known in wildlife parasites, and are needed to model transmission meaningfully (Grear et al. 2013; McCallum et al. 2017). In the meantime, reducing stress and associated behaviours (e.g., dispersal and increased conspecific contact) that may increase parasite transmission in recently translocated animals should be a priority (Aiello et al. 2014; Dickens et al. 2010), as prior studies of *T. adelaidensis* exemplify (Ebrahimi and Bull 2014a, 2014b).

### 4.5 Conclusion

With the potential to cause population decline, the consideration of parasites as part of wildlife translocations is paramount, and provides a practical context in which to study host–parasite ecology and evolution. To our knowledge, this study is the first to use genome‐wide single nucleotide polymorphisms to trace parasite transmission in a wildlife translocation. In doing this, we provide an example of the capability of increasingly accessible technology to help fill knowledge gaps concerning an understudied but ecologically important guild. Specifically, this study has given us a detailed understanding of the consequences of introducing parasites of the same species but differing genetic lineages during the population augmentation of a non‐social host with low vagility. In observing parasite inter‐population genetic structure and slow transmission following translocation in this system, we conclude that the interplay between parasite biology and that of the host is likely to determine the speed at which parasites of different origins transfer to non‐local hosts when they are mixed. A deeper understanding of parasite identity, biology and transmission afforded by this kind of study will inform management that minimises the parasite‐related risks of translocating wildlife hosts while retaining parasite diversity and host–parasite relationships that contribute to ecosystem function.

## Author Contributions

Bonnie T. Derne designed and performed the research, analysed the data, and wrote the manuscript. Stephanie S. Godfrey analysed the data, designed the research, and wrote the manuscript. Mark N. Hutchinson and Philip Weinstein designed the research and wrote the manuscript. Mike G. Gardner designed the research, provided advice on data analysis, and wrote the manuscript.

## Disclosure

Benefits Generated: This research addresses a priority concern, the conservation of the IUCN listed host organism studied, as well as that of its symbionts.

## Conflicts of Interest

The authors declare no conflicts of interest.

## Supporting information


**Appendix S1:** mec70121‐sup‐0001‐Supinfo.zip.
**Table S1:** Analysis of deviance with Type II Wald chi‐square tests of a linear mixed model (using gamma distribution with a log link) examining *Ophiomegistus michaeli* pairwise relatedness as a response to time between collection and host identity (same host or different host). Random factors for the model are: Parasite individual identity, host individual identity. *denotes a significant *p* value (alpha = 0.05).
**Table S2:** Analysis of deviance with Type II Wald chi‐square tests of a linear mixed model examining *Pharyngodon wandillahensis* pairwise relatedness as a response to time between collection and host identity (same host or different host). Random factors for the model are: Parasite individual identity, host individual identity. *denotes a significant *p* value (alpha = 0.05).
**Figure S1:** Percentage of total variance in *Ophiomegistus michaeli* SNPs represented by each dimension in the PCoA.
**Figure S2:** Informativeness of principal components (PCs) derived from PCA of *Ophiomegistus michaeli* genotype data.
**Figure S3:** Bayesian information criterion for each value of *K* during cluster identification in *Ophiomegistus michaeli* genotypes preceding DAPC.
**Figure S4:** (a) Discriminant analysis of principal components (DAPC) of 102 genotyped *Ophiomegistus michaeli* mite individuals where *K* = 3. Cluster names indicate the population origin of the *Tiliqua adelaidensis* hosts. (b) Comparison of group membership based on host origin (rows) and the three clusters (columns) inferred by adegenet's cluster identification algorithm for 102 Ophiomegistus michaeli genotypes. Size of squares represents the number of mite individuals within the intersect of a given sampling group (based on host origin and treatment), and the cluster inferred by DAPC.
**Figure S5:** Cluster membership probabilities for genotyped *Ophiomegistus michaeli* mites assigned by STRUCTURE. Labels indicate groups based on the *Tiliqua adelaidensis* host's population of origin and whether or host was in an experimental enclosure during the translocation (‘Burra mixed’, ‘Jamestown mixed’, ‘Clare mixed’, ‘Unknown mixed’) or was not translocated nor mixing with translocated conspecifics (‘Burra’, ‘Jamestown’, ‘Clare’). (a) All genotyped individuals (*n* = 102) are split into three most likely genetic groups (*K* = 3) (blue, purple and orange). (b) first subset (*n* = 58), (depicted in blue in a) is further separated into two most likely groups (*K* = 2) and; (c) second subset (*n* = 28), (depicted in purple in a) is further separated into three most likely genetic groups (*K* = 3) and; (d) third subset (*n* = 16) (depicted in orange in a) is further separated into two most likely genetic groups (*K* = 2).
**Figure S6:** Percentage of total variance in *Pharyngodon wandillahensis* SNPs represented by each dimension in the PCoA.
**Figure S7:** Informativeness of principal components (PCs) derived from PCA of *Pharyngodon wandillahensis* genotype data.
**Figure S8:** Bayesian information criterion for each value of K during cluster identification in *Pharyngodon wandillahensis* genotypes preceding DAPC. *K* = 3, BIC = 426.7175, 423.8469, 422.5, *K* = 10, BIC = 416.9269.
**Figure S9:** (a) Discriminant analysis of principal components (DAPC) of genotyped 147 *Pharyngodon wandillahensis* nematode individuals where *K* = 3. Cluster names indicate the population origin of the *Tiliqua adelaidensis* hosts. (b) Comparison of group membership based on host origin and the clusters inferred by adegenet's cluster identification algorithm for 147 *Pharyngodon wandillahensis* genotypes. Size of squares represents the number of individuals within the intersect of a given sampling group (based on host origin and treatment), and the cluster inferred by DAPC.
**Figure S10:** Cluster membership probabilities for genotyped *Pharyngodon wandillahensis* nematodes assigned by STRUCTURE. Labels indicate groups based on the *T. adelaidensis* host's population of origin and whether or host was in an experimental enclosure during the translocation (‘Burra mixed’, ‘Jamestown mixed’, ‘Clare mixed’) or was not translocated nor mixing with translocated conspecifics (‘Burra’, ‘Jamestown’, ‘Peterborough’). (a) All genotyped individuals (*n* = 147) are split into two genetic groups (*K* = 2) (blue and orange) (b) first subset (*n* = 104) (depicted in blue in a) is further separated into three most likely groups (*K* = 3) and; (c) second subset (*n* = 43) (depicted in orange in a) is further separated into two most likely genetic groups (*K* = 2).

## Data Availability

All raw SNP genotype data, sample metadata and analysis scripts for this study are accessible at the following: https://doi.org/10.25451/flinders.28602965. [N.B. Available at https://figshare.com/s/001315b0c7c1b0f0a8fe for reviewing purposes].
