# Supplementary material for: Mixing in Moderation: Slow Transmission of Non‐Local Macroparasites Following a Population Augmentation of an Endangered Australian Skink
Source: Mol Ecol. 2025 Sep 30;34(20):e70121. doi: 10.1111/mec.70121 (PMC12530289; doi:10.1111/mec.70121)
Supplement: Supplementary file 1 — Appendix S1: mec70121‐sup‐0001‐Supinfo.zip. Table S1: Analysis of deviance with Type II Wald chi‐square tests of a linear mixed model (using gamma distribution with a log link) examining Ophiomegistus michaeli pairwise relatedness as a response to time between collection and host identity (same host or different host). Random factors for the model are: Parasite individual identity, host individual identity. *denotes a significant p value (alpha = 0.05). Table S2: Analysis of deviance with Type II Wald chi‐square tests of a linear mixed model examining Pharyngodon wandillahensis pairwise relatedness as a response to time between collection and host identity (same host or different host). Random factors for the model are: Parasite individual identity, host individual identity. *denotes a significant p value (alpha = 0.05). Figure S1: Percentage of total variance in Ophiomegistus michaeli SNPs represented by each dimension in the PCoA. Figure S2: Informativeness of principal components (PCs) derived from PCA of Ophiomegistus michaeli genotype data. Figure S3: Bayesian information criterion for each value of K during cluster identification in Ophiomegistus michaeli genotypes preceding DAPC. Figure S4: (a) Discriminant analysis of principal components (DAPC) of 102 genotyped Ophiomegistus michaeli mite individuals where K = 3. Cluster names indicate the population origin of the Tiliqua adelaidensis hosts. (b) Comparison of group membership based on host origin (rows) and the three clusters (columns) inferred by adegenet's cluster identification algorithm for 102 Ophiomegistus michaeli genotypes. Size of squares represents the number of mite individuals within the intersect of a given sampling group (based on host origin and treatment), and the cluster inferred by DAPC. Figure S5: Cluster membership probabilities for genotyped Ophiomegistus michaeli mites assigned by STRUCTURE. Labels indicate groups based on the Tiliqua adelaidensis host's population of origin and whethe [file MEC-34-e70121-s001.zip › 3_PostTranslocationParasiteTransmission_SupportingInfo_MERevisions_280925 (1).docx]

#### Mixing in moderation: slow transmission of non-local macroparasites following a population augmentation of an endangered Australian skink

## **Supporting Information**

### **Materials & Methods**

### **Single nucleotide polymorphism identification by DArTSeq**

Extraction of DNA from whole nematodes and mites was conducted using protocols developed by Diversity Array Technology Pty Ltd (DArTseq) (Kilian et al., 2012). Following optimisation, DNA was digested using the restriction enzymes PstI and SphI and then labelled with sample-specific adaptors and sequenced with short-read Next Generation Sequencing (Illumina) at a sequencing depth of 0.8 million reads, as per the proprietary in-house pipelines of DArTseq (Kilian et al., 2012). One third of DNA samples were processed twice (with different adapters and independent allelic calls) as technical replicates to ensure reproducibility. A set of raw ‘sequence tags’ of approximately 75 bp in length were produced and quality filtered. Various proprietary filters were then applied to identify sequence tags that contained a reliable SNP marker.

### **Single nucleotide polymorphism filtering**

These DArTseq data were imported into the R (R Core Team, 2020 (R Core Team, 2022) package dartR (Gruber et al., 2018), converted to genlight format (Jombart et al., 2008) and filtered before subsequent analysis. The first filtering step removed individuals and loci for which the reproducibility (averaged over two allelic states) fell below 100%, and also removed monomorphic loci. Loci that had over 25% of missing data across individuals were removed, and then any secondary loci on sequence tags were removed. Next, individuals that had over 25% of missing data across loci were removed. Loci pairs with a Hamming distance of less than 0.2 were removed to reduce the possibility of sequencing error being confused with a different locus. Filters to remove any loci not in Hardy-Weinberg equilibrium were applied. Loci in linkage disequilibrium were retained in the interests of retaining the maximum number of SNPs for population-level differentiation. An additional filter to remove loci with minor allele frequencies less than 1% was applied for subsequent relatedness analysis.

### **Principal coordinates analysis (PCoA)**

Genetic similarity between individual parasites from different populations was examined using principal coordinates analysis (PCoA) ordination (Gower, 1966) in dartR, where individuals were entities and SNP loci were the attributes. A plot placing each individual by the two most informative dimensions – the loci which explained the greatest proportion of the total variation between individuals – was produced to visualise this ordination. Dimensions were considered informative when they explained more than the average of original variables.

### **Discriminant analysis of principal components (DAPC)**

Another non model-based method for grouping individuals based on genetic similarity was applied by performing cluster identification and DAPC with the R package adegenet (Jombart et al., 2008). DAPC focuses on between-group variability of genetically related individuals, which may enable clearer identification of clusters, in contrast to principal component analysis (PCA) or PCoA, which summarise overall variability between individuals (Jombart et al., 2010). First, data were transformed by PCA and all principal components (PCs) were retained. Possible clusters were identified, and Bayesian information criterion (BIC) was used to choose an optimal value of K (a biologically plausible number of clusters that maximises variation). DAPC was then performed using only a subset of the most informative PCs in order to balance between sufficient informativeness with overfitting and instability of the membership probabilities returned(Jombart & Collins, 2015). All discriminant functions were retained since the number of possible clusters were low.

### **Bayesian cluster analysis with STRUCTURE**

The software STRUCTURE 2.3.4 (Pritchard et al., 2000) was used to conduct Bayesian model-based cluster analysis, as an alternative way to examine whether or not SNP genotypes clustered by host population of origin, and to identify any evidence of allopatric parasite transmission between hosts of different origins within the translocation. Using GNU Parallel (Tange, 2018), 20 replicates for each value of k (number of populations) between 1–10 were run with different random seeds, as recommended by Evanno et al. (2005). Each run consisted of 100,000 burn-in iterations (as deemed sufficient by convergence of values of summary statistics (Porras-Hurtado et al., 2013) and 100,000 Markov Chain Monte Carlo (MCMC) repetitions. The population-specific prior was selected (POPALPHA=1) and alpha was set to 1/K, where K was the assumed number of populations(Wang, 2017). All other extra parameters were left at default options, notably Admixture models were used, and the correlated allele frequencies option was selected (Falush et al., 2003). In the absence of a genetic map, linkage models were not used.

The likelihood of each value of K, L(K), given the data, was averaged across replicates and plotted by Structure Harvester (Earl & VonHoldt, 2012). Unless the plot of L(K) clearly indicated a K value of 1 (i.e. there was no or little increase in likelihood with higher values of K), we used the complementary value of delta K to determine the most likely value of K outlined by Evanno et al. (2005). Where delta K was highest for K=2 or more, members of these identified sub-clusters were identified by CLUMPAK (Kopelman et al., 2015)using the greedy algorithm. An individual was considered a member of the cluster for which it had the highest estimated probability of membership, as compared to a higher probability threshold (e.g. 0.6 used by Coulson et al. (2008). Delta K reliably detects the uppermost hierarchical level of population structure, though this may not reflect the true value of K (Evanno et al., 2005; Janes et al., 2017) Therefore, hierarchical structure analysis was conducted for each identified cluster (Coulon et al., 2008; Janes et al., 2017). This process involved further STRUCTURE runs and analysis of results, as outlined above for each identified sub-cluster. Sub-clusters of less than 4 individuals identified during hierarchical analysis were considered as one cluster and not further analysed. The estimated membership probabilities of different clusters for each individual were visualised using CLUMPAK (Kopelman et al. 2015).

### **Mite relatedness**

Filtered SNP data for 102 genotyped mites were imported into the program COANCESTRY 1.0.1.9 (Wang, 2011) in order to estimate relatedness between individual mites. Simulations of mite genotypes using the allelic frequencies derived from the genotype data and missing data rates at each locus were compared to expected relatedness levels for each relationship class (e.g. parent-offspring, full-siblings, unrelated). The estimator providing the highest correlation between the estimated and expected relatedness values of dyads, in this case the dyad maximum likelihood estimator (Milligan, 2003), was used to estimate relatedness between sampled individuals (herein referred to as relatedness). Relatedness between all mites in the dataset was reduced to a set of dyads between mites found on hosts in the same enclosures.

| **Estimation method** | **Trio-ML** | **Wang** | **Lynch-Li** | **Lynch-Ritland** | **Ritland** | **Queller-Goodnight** | **DyadML** |
| --- | --- | --- | --- | --- | --- | --- | --- |
| **Correlation Coefficient, r(398)** | .91 | .86 | .86 | .87 | .78 | .87 | .92 |
| **p** | p<.001 | p<.001 | p<.001 | p<.001 | p<.001 | p<.001 | p<.001 |

**Table S1. Correlation coefficients between the estimated and expected relatedness of 400 *Ophiomegistus michaeli* dyads using the seven relatedness estimation methods implemented by COANCESTRY** (Wang, 2011)**.**

### **Nematode relatedness**

Filtered SNP data were imported into the program PolyRelatedness 1.8 (Huang et al., 2014) in order to estimate relatedness between individual nematodes as *Pharyngodon wandillahensis*, as members of the order Oxyurida, would be haplo-diploid (Adamson, 1989). Nematodes which were heterozygotes for two or less of 358 filtered loci were considered to be haploid individuals (23/147) and entered as such. Simulations of haplodiploid nematode genotypes using the allele frequencies derived from the empirical data showed that the Ritland’s estimator of relatedness with Huang’s correction (Huang et al., 2015) was most closely correlated with expected relatedness values for different relationship classes. This estimator was therefore used to estimate relatedness between sampled individuals. Unlike the dyad ML estimator used for mite relatedness, this estimator had a scale range between 1 and -1, where negative relatedness indicated pairs that were less related than average. As with relatedness for mites, relatedness for nematodes pairs was sorted into enclosures.

### **Results**

### **Principal coordinate analysis and discriminant analysis of principal components of *Ophiomegistus michaeli* and *Pharyngodon wandillahensis* SNPs**

**Figure S1. Percentage of total variance in *Ophiomegistus michaeli* SNPs represented by each dimension in the PCoA.**

##### **Figure S2. Informativeness of principal components (PCs) derived from PCA of *Ophiomegistus michaeli* genotype data.**

##### **Figure S3. Bayesian information criterion for each value of K during cluster identification in *Ophiomegistus michaeli* genotypes preceding DAPC.**


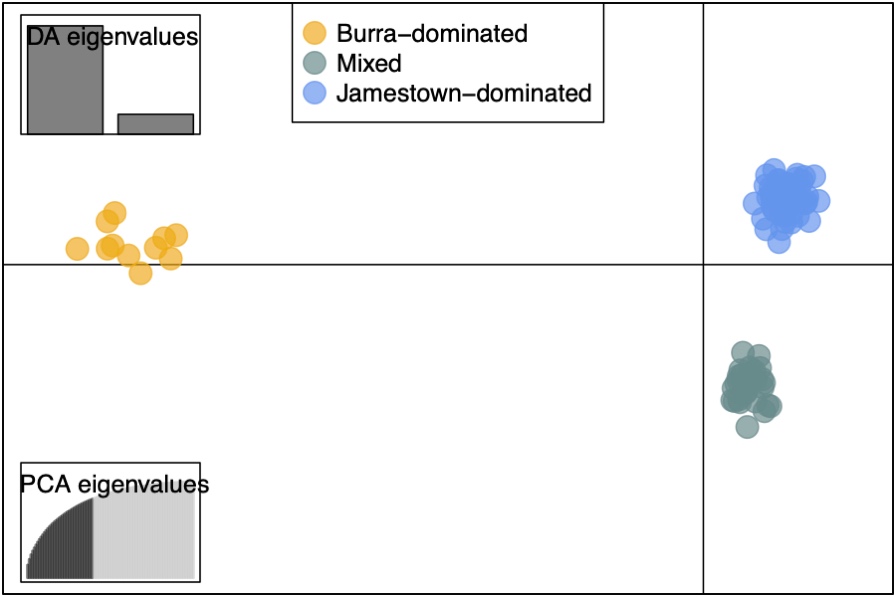

**a)**

**b)**

**Figure S4. a) Discriminant analysis of principal components (DAPC) of 102 genotyped *Ophiomegistus michaeli* mite individuals where K=3.** Note: Cluster names indicate the population origin of the *Tiliqua adelaidensis* hosts**. b) Comparison of group membership based on host origin (rows) and the three clusters (columns) inferred by adegenet’s cluster identification algorithm for 102 *Ophiomegistus michaeli* genotypes.** Note: Size of squares represents the number of mite individuals within the intersect of a given sampling group (based on host origin and treatment), and the cluster inferred by DAPC.

##### **Figure S5. Cluster membership probabilities for genotyped *Ophiomegistus michaeli* mites assigned by STRUCTURE.** Note: Labels indicate groups based on the *Tiliqua adelaidensis* host’s population of origin and whether or host was in an experimental enclosure during the translocation (‘Burra mixed’, ‘Jamestown mixed’, ‘Clare mixed’, ‘Unknown mixed’) or was not translocated nor mixing with translocated conspecifics (‘Burra’, ‘Jamestown’, ‘Clare’). a**) All genotyped individuals (n=102) are split into three most likely genetic groups** (K=3) (blue, purple and orange). **b) first subset** (n=58), (depicted in blue in a)) is further separated into two most likely groups (K=2) and; **c) second subset** (n=28), (depicted in purple in a)) is further separated into three most likely genetic groups (K=3) and; **d) third subset** (n=16) (depicted in orange in a)) is further separated into two most likely genetic groups (K=2).

##### **Figure S6. Percentage of total variance in *Pharyngodon wandillahensis* SNPs represented by each dimension in the PCoA.**

##### **Figure S7. Informativeness of principal components (PCs) derived from PCA of *Pharyngodon wandillahensis* genotype data.**

**Figure S8. Bayesian information criterion for each value of K during cluster identification in *Pharyngodon wandillahensis* genotypes preceding DAPC. Note: K=3, BIC=426.7175, 423.8469, 422.5, K=10, BIC=416.9269**


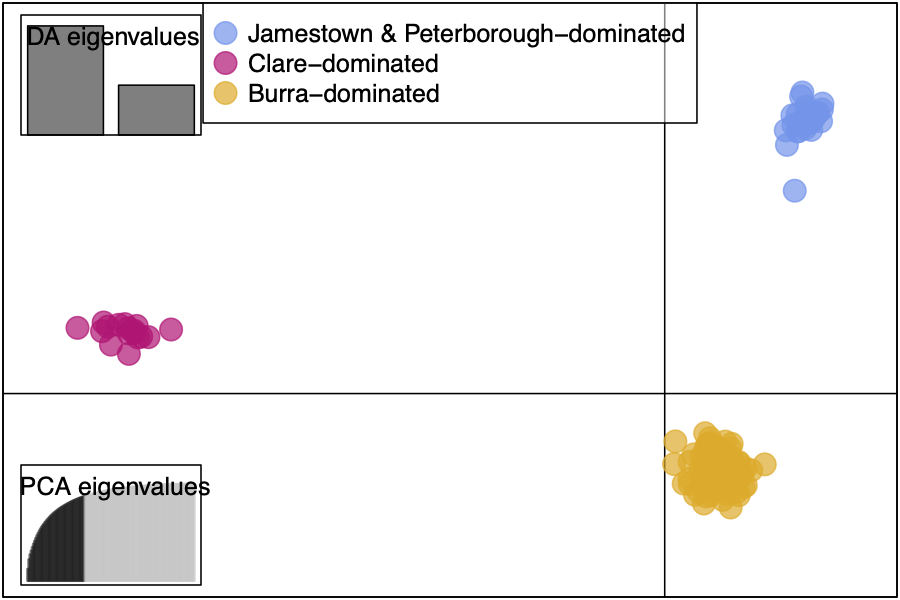


**a)**

**b)**

**Figure S9. a) Discriminant analysis of principal components (DAPC) of genotyped 147 *Pharyngodon wandillahensis* nematode individuals where K=3**. Note: Cluster names indicate the population origin of the *Tiliqua adelaidensis* hosts. **b) Comparison of group membership based on host origin and the clusters inferred by adegenet’s cluster identification algorithm for 147 *Pharyngodon wandillahensis* genotypes.** Note: Size of squares represents the number of individuals within the intersect of a given sampling group (based on host origin and treatment), and the cluster inferred by DAPC.

##### **Figure S10. Cluster membership probabilities for genotyped *Pharyngodon wandillahensis* nematodes assigned by STRUCTURE. Labels indicate groups based on the *T. adelaidensis* host’s population of origin and whether or host was in an experimental enclosure during the translocation (‘Burra mixed’, ‘Jamestown mixed’, ‘Clare mixed’) or was not translocated nor mixing with translocated conspecifics (‘Burra’, ‘Jamestown’, ‘Peterborough’). a) All genotyped individuals (n=147) are split into two genetic groups (K=2) (blue and orange) b) first subset (n=104) (depicted in blue in a)) is further separated into three most likely groups (K=3) and; c) second subset (n=43) (depicted in orange in a)) is further separated into two most likely genetic groups (K=2).**

**Table S2. Analysis of deviance with Type II Wald chi-square tests of a linear mixed model (using gamma distribution with a log link) examining *Ophiomegistus michaeli* pairwise relatedness as a response to time between collection and host identity (same host or different host). Random factors for the model are: Parasite individual identity, host individual identity. * denotes a significant p value (alpha=0.05)**

| Model focus |  | | | |
| --- | --- | --- | --- | --- |
| Effect of time between collection and host identity | **Fixed effects** | **𝝌^2^** | **Df** | **Pr(>𝝌^2^)** |
|  | Same/different host | 35.619 | 1 | <0.001 * |
|  | Days between mite collection | 1.886 | 1 | 0.170 |
|  | Sampling season | 0.518 | 2 | 0.772 |
|  | Same/different host : Days between collection | 0.767 | 1 | 0.3811 |

**Table S3. Analysis of deviance with Type II Wald chi-square tests of a linear mixed model examining *Pharyngodon wandillahensis* pairwise relatedness as a response to time between collection and host identity (same host or different host). Random factors for the model are: Parasite individual identity, host individual identity. * denotes a significant p value (alpha=0.05)**

| Model focus |  | | | |
| --- | --- | --- | --- | --- |
| Effect of time between collection and host identity | **Fixed effects** | **𝝌^2^** | **Df** | **Pr(>𝝌^2^)** |
|  | Same/different host | 206.868 | 1 | <0.001* |
|  | Days between nematode collection | 0.582 | 1 | 0.446 |
|  | Sampling season | 0.196 | 2 | 0.907 |
|  | Same/different host : Days between collection | 14.127 | 1 | <0.001* |

Adamson, M. L. (1989). Evolutionary Biology of the Oxyurida (Namatoda): Biofacies of a Haplodiploid Taxon. *Advances in Parasitology*, *28*(C), 175–228. https://doi.org/10.1016/S0065-308X(08)60333-4

Coulon, A., Fitzpatrick, R., Bowman, B., Stith, B. M., Makarewich, A., Stenzler, L. M., & Lovette, I. J. (2008). Congruent population structure inferred from dispersal behaviour and intensive genetic surveys of the threatened Florida scrub-jay (Aphelocoma cœrulescens). *Molecular Ecology*, *17*(7), 1685–1701. https://doi.org/10.1111/j.1365-294X.2008.03705.x

Earl, D., & VonHoldt, B. (2012). STRUCTURE HARVESTER : a website and program for visualizing STRUCTURE output and implementing the Evanno method. *Conservation Genetics Resources*, *4*, 359–361. https://doi.org/10.1007/s12686-011-9548-7

Evanno, G., Regnaut, S., & Goudet, J. (2005). Detecting the number of clusters of individuals using the software STRUCTURE : a simulation study. *Molecular Ecology*, *14*(18), 2611–2620. https://doi.org/10.1111/j.1365-294X.2005.02553.x

Falush, D., Stephens, M., & Pritchard, J. K. (2003). Inference of Population Structure Using Multilocus Genotype Data: Linked Loci and Correlated Allele Frequencies. *Genetics*, *164*(4), 1567–1587.

Gower, J. C. (1966). Some distance properties of latent root and vector methods used in multivariate analysis. *Biometrika*, *53*(3–4), 325–338.

Huang, K., Guo, S. T., Chen, S. T., Qi, X. G., Zhang, P., & Li, B. G. (2015). A maximum-likelihood estimation of pairwise relatedness for autopolyploids. *Heredity*, *114*(2), 133–142. https://doi.org/10.1038/hdy.2014.88

Huang, K., Ritland, K., Guo, S., Shattuck, M., & Li, B. (2014). A pairwise relatedness estimator for polyploids. *Molecular Ecology Resources*, *14*(4), 734–744. https://doi.org/10.1111/1755-0998.12217

Janes, J. K., Malenfant, M., Andrew, R. L., Miller, J. M., Dupuis, J. R., Gorrell, J. C., & Cullingham, C. I. (2017). The K=2 conundrum. *Molecular Ecology*, *26*(14), 3594–3602. https://doi.org/10.1111/mec.14187

Jombart, T., & Collins, C. (2015). *A tutorial for Discriminant Analysis of Principal Components (DAPC) using adegenet 2.0.0*. http://adegenet.r-forge.r-project.org/files/tutorial-dapc.pdf

Jombart, T., Devillard, S., & Balloux, F. (2010). Discriminant analysis of principal components : a new method for the analysis of genetically structured populations. *BMC Genetics*, *11*, 94.

Jombart, T., Lyon, D., & Biome, L. De. (2008). adegenet : a R package for the multivariate analysis of genetic markers. *Bioinformatics*, *24*(11), 1403–1405. https://doi.org/10.1093/bioinformatics/btn129

Kilian, A., Wenzl, P., Huttner, E., Carling, J., Xia, L., Caig, V., Heller-uszynska, K., Jaccoud, D., Hopper, C., Aschenbrenner-kilian, M., Evers, M., Peng, K., Cayla, C., Hok, P., & Uszynski, G. (2012). Diversity Arrays Technology : A Generic Genome Profiling Technology on Open Platforms. In F. Pompanon & A. Bonin (Eds.), *Data production and analysis in population genomics, Methods in Molecular Biology* (Vol. 888, pp. 67–89). Springer Science+Business Media. https://doi.org/10.1007/978-1-61779-870-2

Kopelman, N. M., Mayzel, J., Jakobsson, M., Rosenberg, N. A., & Mayrose, I. (2015). CLUMPAK: a program for identifying clustering modes and packaging population structure inferences across K. *Molecular Ecology Resources*, *15*(5), 1179–1191. https://doi.org/10.1111/1755-0998.12387

Milligan, B. G. (2003). Maximum-likelihood estimation of relatedness. *Genetics*, *163*(3), 1153–1167.

Porras-Hurtado, L., Ruiz, Y., Santos, C., Phillips, C., Carracedo, A., & Lareu, M. V. (2013). An overview of STRUCTURE : applications , parameter settings , and supporting software. *Frontiers in Genetics*, *4*(98), 1–13. https://doi.org/10.3389/fgene.2013.00098

Pritchard, J. K., Stephens, M., & Donnelly, P. (2000). Inference of Population Structure Using Multilocus Genotype Data. *Genetics*, *155*(2), 945–959.

R Core Team. (2020). R: A language and environment for statistical computing. In *R Foundation for statistical computing*.

Tange, O. (2018). *GNU Parallel 2018*. Ole Tange. https://doi.org/10.5281/zenodo.1146014

Wang, J. (2011). COANCESTRY: a program for simulating, estimating and analysing relatedness and inbreeding coefficients. *Molecular Ecology Resources*, *11*(1), 141–145. https://doi.org/10.1111/j.1755-0998.2010.02885.x

Wang, J. (2017). The computer program STRUCTURE for assigning individuals to populations : easy to use but easier to misuse. *Molecular Ecology Resources*, *17*(5), 981–990. https://doi.org/10.1111/1755-0998.12650
